# Supplementary material for: Genetic Diversity, Population Structure, and Environmental Adaptation Signatures of Chinese Coastal Hard-Shell Mussel Mytilus coruscus Revealed by Whole-Genome Sequencing
Source: Int J Mol Sci. 2023 Sep 4;24(17):13641. doi: 10.3390/ijms241713641 (PMC10488143; doi:10.3390/ijms241713641)
Supplement: Supplementary file 1 [file ijms-24-13641-s001.zip › Supplementary Information.pdf]

Supplementary Information

Genetic Diversity, Population Structure, and Environmental Adaptation Signatures of Chinese Coastal Hard-Shell Mussel *Mytilus coruscus* Revealed by Whole-Genome Sequencing

Feng Guo [1,†], Yingying Ye [1,†], Kecheng Zhu [2], Shuangrui Lin [1], Yuxia Wang [3], Zhenyu Dong [3], Ronghui Yao [3], Hongfei Li [1], Weifeng Wang [3], Zhi Liao [3], Baoying Guo [1,3,*] and Xiaojun Yan [1,3,*]

[1] National Engineering Research Center for Marine Aquaculture, Zhejiang Ocean University, Zhoushan 316022, China; agsguo@163.com (F.G.); yeyy@zjou.edu.cn (Y.Y.); citric-acid@foxmail.com (S.L.); lhf612@126.com (H.L.)
[2] Key Laboratory of South China Sea Fishery Resources Exploitation and Utilization, Ministry of Agriculture and Rural Affairs, South China Sea Fisheries Research Institute, Chinese Academy of Fishery Sciences, Guangzhou 510300, China; zhukecheng@scsfri.ac.cn
[3] Marine Science and Technology College, Zhejiang Ocean University, Zhoushan 316022, China; wangyuxia97@163.com (Y.W.); dongzhenyu2021@163.com (Z.D.); 17662452562@163.com (R.Y.); wangwf@zjou.edu.cn (W.W.); liaozhi@zjou.edu.cn (Z.L.)
* Correspondence: guobaoying@zjou.edu.cn (B.G.); yanxj@zjou.edu.cn (X.Y.)
† These authors contributed equally to this work.

Table. S1 The details of sampling

Sample Site	Abbr	Coordinate	Sampling Date	Sample Size
Dalian	DL	38°92′ **N, 121°62’ E**	2020.11	10
Qingdao	QD	35°12′ **N, 120°30’ E**	2020.12	10
Lianyungang	LYG	34°60′ **N, 119°22’ E**	2021.02	10
Zhoushan	ZS	30°12′ **N, 121°62’ E**	2021.02	10
Xiangshan	XS	29°14′ **N, 121°58’ E**	2021.03	10
Yuhuan	YH	28°14′ **N, 121°24’ E**	2021.03	10
Taishan Island	TS	26°57′ **N, 120°47’ E**	2021.04	10
Zhangzhou	ZZ	24°52′ **N, 117°65’ E**	2021.04	10
Total				80

Table. S2 Population differentiation index (F_{ST}) between two of the eight geographical populations

	QD	LYG	ZS	XS	YH	TS	ZZ
DL	-0.001	0.010	-0.001	-0.002	-0.002	-0.002	0.002
QD		0.011	0.001	-0.001	-0.001	-0.002	0.014
LYG			0.021	0.011	0.012	0.011	0.031
ZS				-0.001	-8.74E-05	-0.0002	0.012
XS					-0.002	-0.002	0.013
YH						-0.002	0.011
TS							0.011

Table. S3 Sample sequencing results and genome mapping rate statistics

Table. S4-S5 The results of the selection signature analysis

Table. S6-S7 GO enrichment results of selected genes in Zhangzhou

Table. S8 Genome-wide nucleotide diversity data in bivalves

Species	Pi	Method	DOI
Mytilus coruscus	0.0032-0.0038	whole-genome sequencing	
Crassostrea ariakensis	0.00033-0.00035	whole-genome sequencing	10.1038/s42003-021-02823-6.
Crassostrea gigas	0.00927	whole-genome sequencing	10.1038/s42003-021-02823-6.
Crassostrea ariakensis	0.0010-0.0012	Whole genome sequencing	10.1111/1755-0998.13556
Mizuhopecten yessoensis	0.000018-0.000023	whole-genome sequencing	10.1016/j.aquaculture.2021.737842

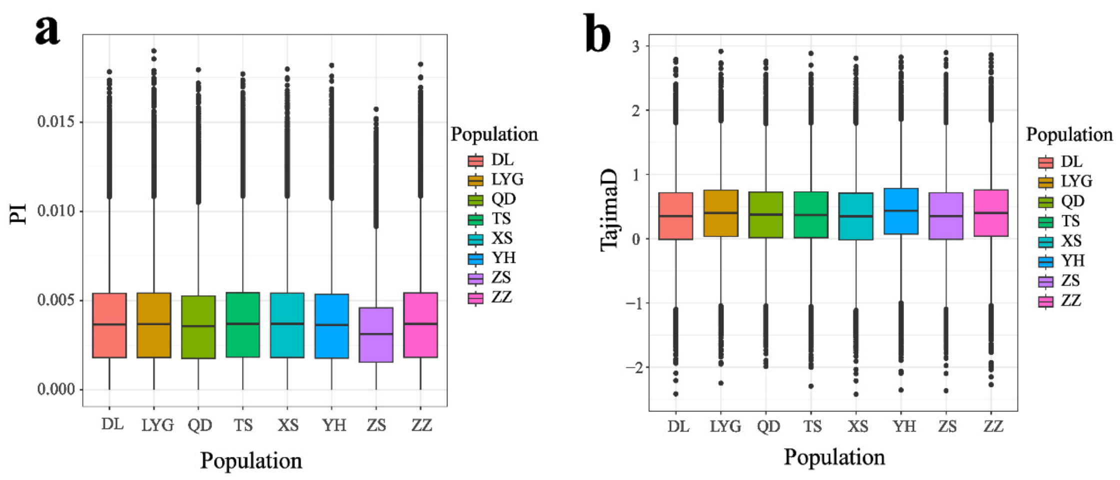

Fig. S1 Nucleotide diversity index and neutrality test results. (a) Nucleotide diversity of the whole genome of hard-shell mussels from eight regions. (b) Tajima's D in the whole genome of eight regional hard-shell mussels.

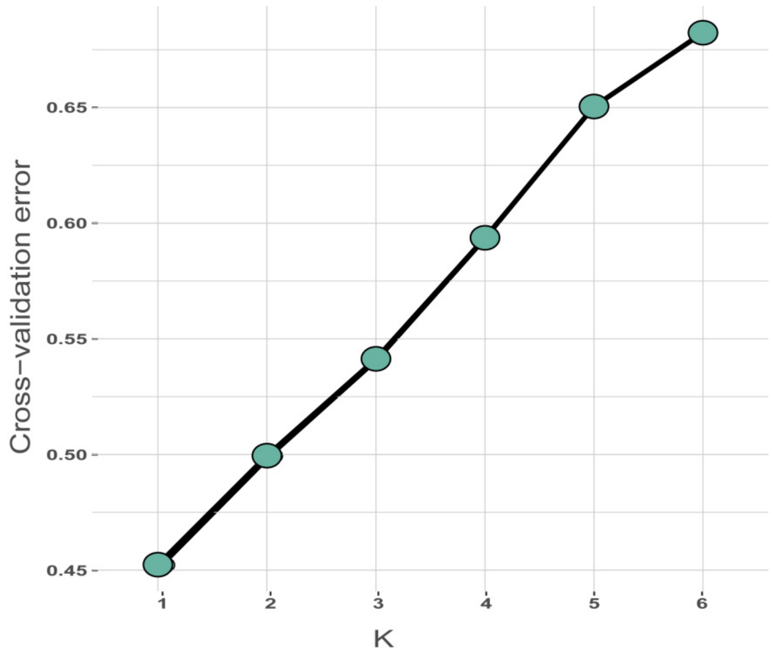

Fig. S2 Relationship curve between K value and Cross-validation error value.

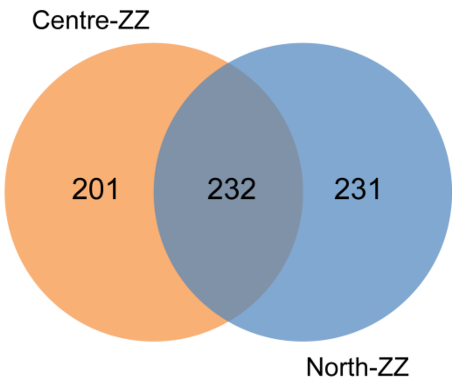

Fig. S3 Overlapping candidate genes in Centre/ZZ and North/ZZ pairs based on Venn diagram.

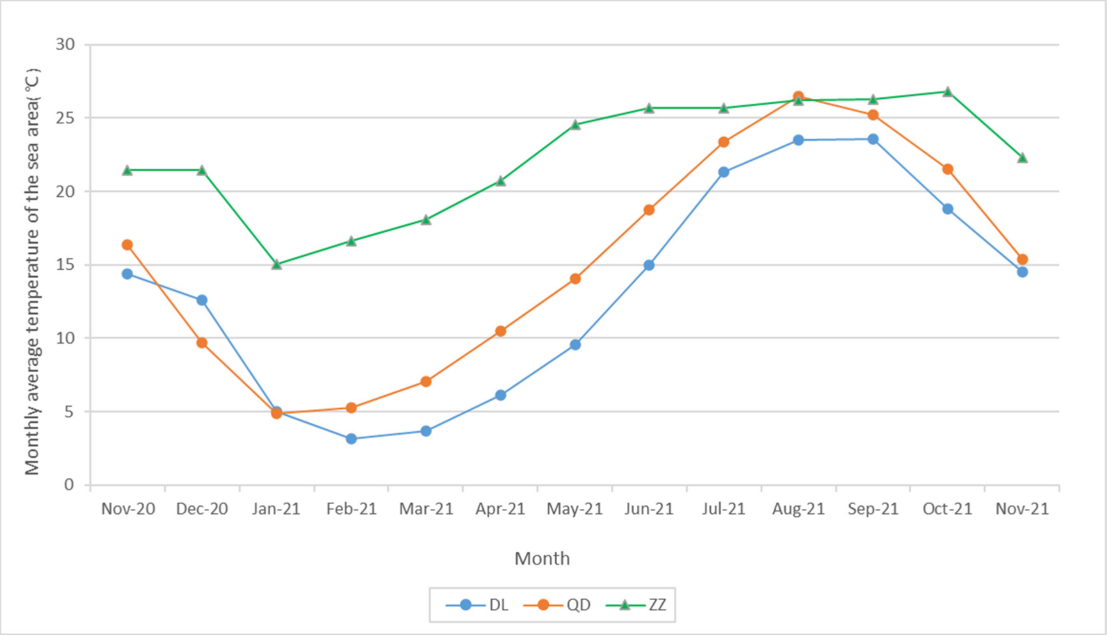

Fig. S4 The monthly average sea surface temperature of three sampling sites (Dalian, Qingdao, and Zhangzhou) was determined through satellite remote sensing from November 2020 to November 2021. Data provided by China's National Science and Technology Resources Sharing Service Platform - National Data Center for Marine Science (http://mds.nmdis.org.cn/).
